# Supplementary material for: Group 1 mGluR stimulation rescues APOE4-mediated translation defects in neurons
Source: Life Sci Alliance. 2025 Nov 11;9(2):e202503287. doi: 10.26508/lsa.202503287 (PMC12614780; doi:10.26508/lsa.202503287)
Supplement: Supplementary file 3 [file LSA-2025-03287_SdataF2.2.pdf]

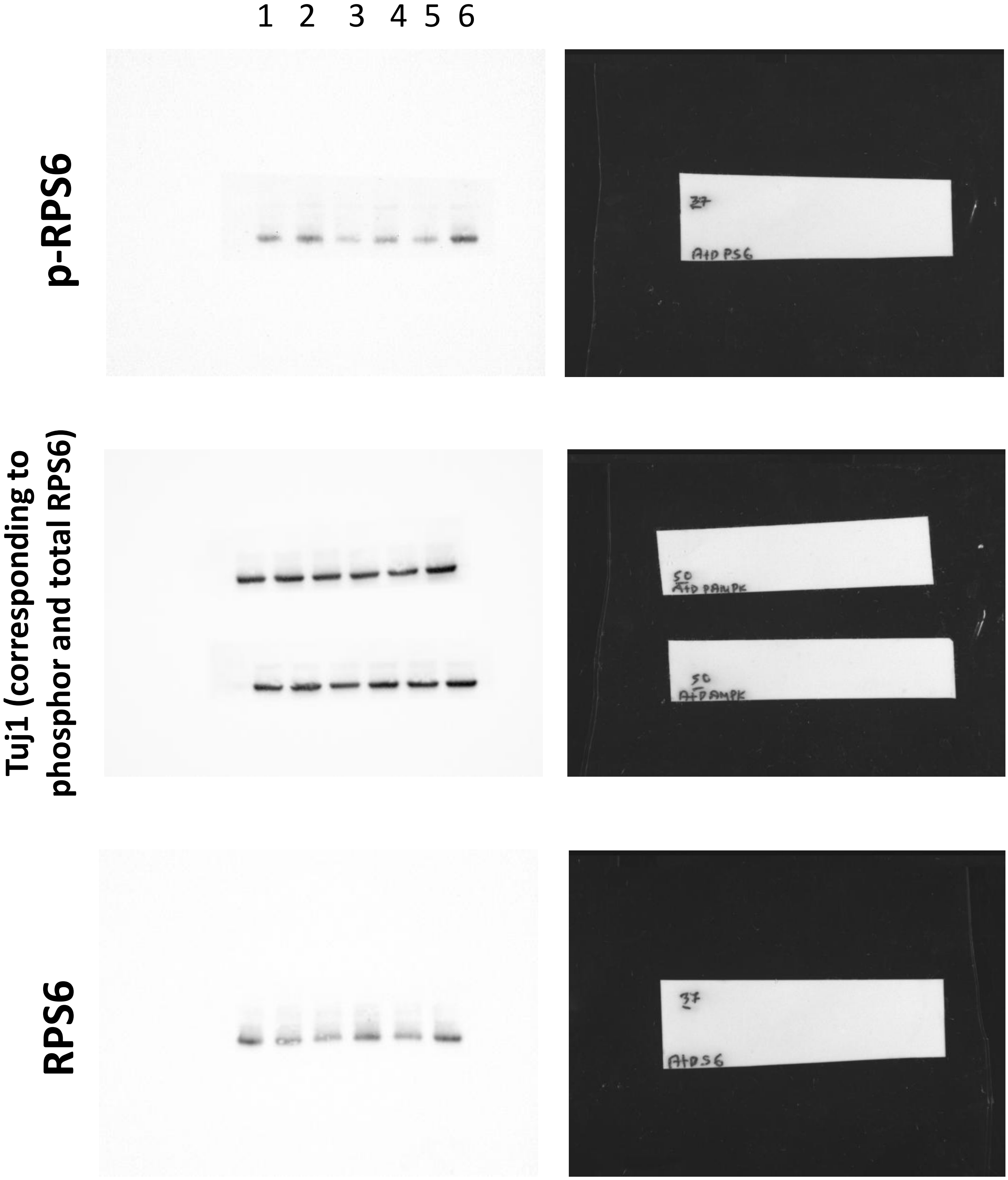

1. Untreated,
2. DHPG-5',
3. APOE4-20',
4. APOE4-20'+DHPG-5',
5. APOE3-20',
6. APOE3-20'+DHPG-5'

Source Data for Figure 2. Original uncropped blots corresponding to Main Figure 2d. The conditions in lanes 1, 2, 3, 4 correspond to the conditions in graph in main figure 2D.

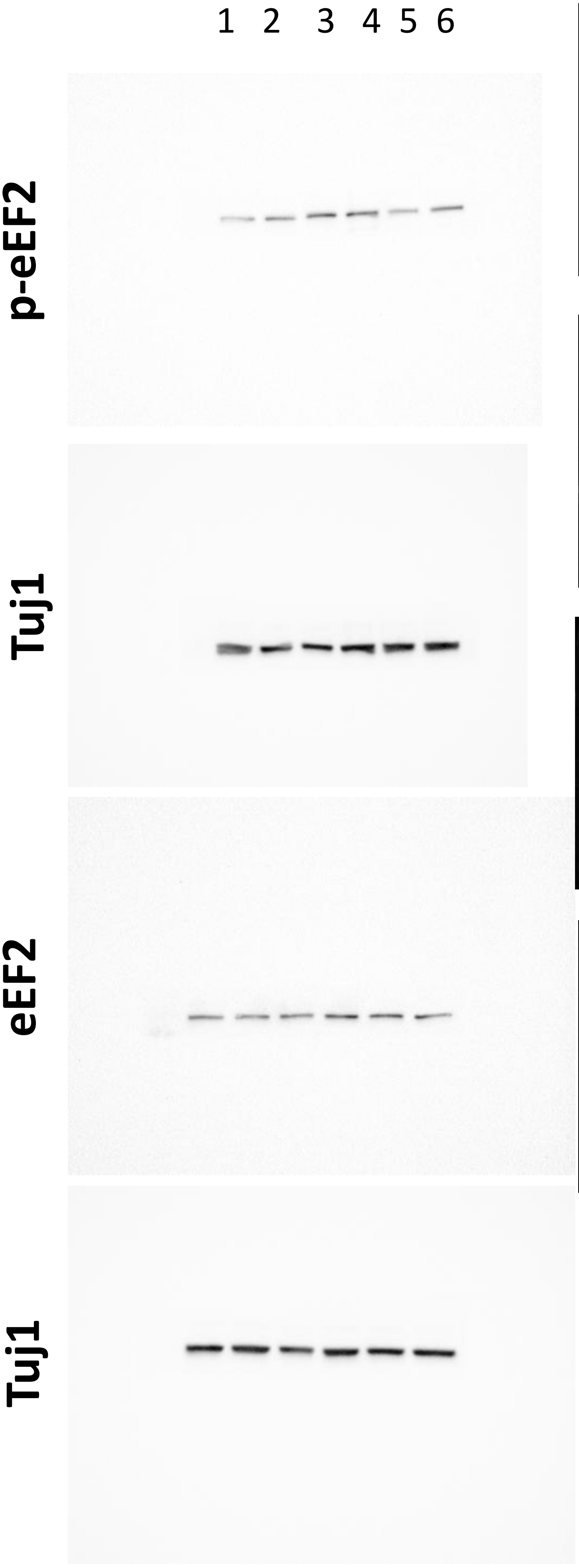

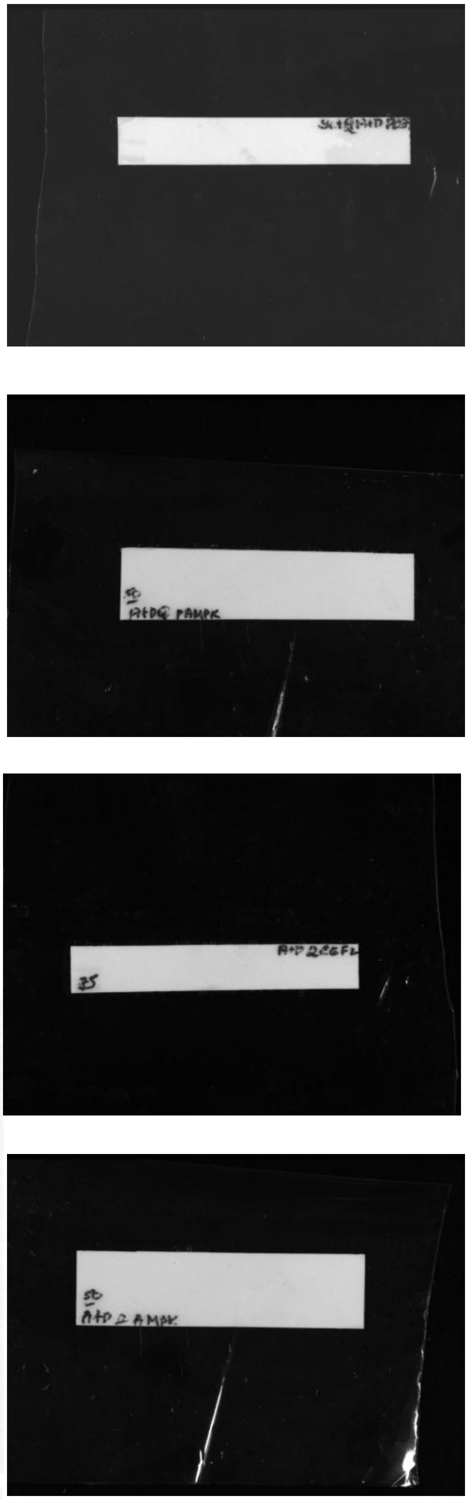

1. Untreated, 2. DHPG-5’, 3. APOE4-20’, 4. APOE4-20’+DHPG-5’, 5. APOE3-20’, 6. APOE3-20’+DHPG-5’

Source Data for Figure 2. Original uncropped blots corresponding to Main Figure 2G. The conditions in lanes 1, 2, 3, 4 correspond to the conditions in graph in main figure 2G.
